# Frailty and Loneliness in Older Adults: A Narrative Review

**DOI:** 10.3390/geriatrics9050119

**Published:** 2024-09-13

**Authors:** Andreea-Cristina Gheorghe, Elena Bălășescu, Ionela Hulea, Gabriela Turcu, Mihai Iustin Amariei, Alin-Victor Covaciu, Cătălina-Andreea Apostol, Melisa Asan, Andrei-Cosmin Badea, Ana-Cristina Angelușiu, Maria-Mirabela Mihailescu-Marin, Daniela Adriana Ion, Roxana Ioana Nedelcu

**Affiliations:** 1 Pathophysiology II Discipline, Faculty of Medicine, “Carol Davila” University of Medicine and Pharmacy, 050474 Bucharest, Romania; 2 Department of Rehabilitation Medicine, Elias University Hospital, 11461 Bucharest, Romania; 3 Department of Dermatology, Colentina Clinical Hospital, 020125 Bucharest, Romania; 4 Department of Medical and Surgical Specialties, “Transilvania” University, 500036 Brasov, Romania

**Keywords:** frailty, loneliness, older people, quality of life, aging, healthcare, patient-centered care

## Abstract

(1) Background: In a society with an advancing aging rate, medical systems are coming under pressure due to an increasing flow of older patients with multiple somatic diseases, exacerbated by their psychological and sociological backgrounds. We aimed to investigate the relationship between frailty and loneliness in older adults and to provide a holistic perspective on these concepts. Our research question was “Is there a link between the loneliness and frailty in older people?” (2) Methods: To assess the link between loneliness and frailty, we conducted a search accessing Index Medicus and PubMed; the timeframe of our research was from 2013 until 2023. Data regarding the study population, as well as loneliness and frailty assessments and approaches, were extracted. (3) Results: A positive relationship between loneliness and the appearance and progression of frailty in older adults is argued for. (4) Conclusions: Frailty and loneliness in older adults are often interconnected and can have a significant impact on their overall well-being. Early identification of frailty by assessing risk factors (including loneliness and/or social isolation) should become a standard of care for older patients. Appropriate combined interventions that effectively address both frailty and loneliness (physical exercises, psychological support, and social engagement) can promote healthier aging, prevent health deterioration, maintain independence, and reduce healthcare costs.

## 1. Introduction

### 1.1. Background

In a society with an advanced aging rate, medical systems are put under pressure due to the increasing flow of older people with multiple somatic diseases, sometimes exacerbated by a fragile psychological background. Maintaining a balance in the body’s functionality for as long as possible requires the diagnosis and periodic assessment of health status at any age, which, due to the various socio-financial and emotional implications, is particularly imperative for people who are in their later years. A positive relationship between loneliness and the appearance and progression of frailty in older people has been argued [1,2,3]. Frailty is potentially preventable, and it seems that it could even be reversed if early diagnosis and appropriate interventions are made [4]. Therefore, knowledge of the associated risk factors is an absolute necessity to slow down, stop, or even reverse the frailty process. All of these are arguments for carrying out our study.

### 1.2. Rationale and Knowledge Gap

Our work aims to fill the knowledge gap regarding the supposed bidirectional link between loneliness and frailty. Despite existing data on frailty and its risk factors and the consequences of loneliness on physical, mental, and emotional health, there is a lack of research in understanding how loneliness may influence frailty and vice versa.

A more refined understanding of loneliness and its impact on cognitive, emotional, and physical health may enhance the effectiveness of clinical practices and public health policies aimed at mitigating loneliness and its associated health risks. By studying the relationship between loneliness and physical frailty, we aimed to offer a holistic perspective on these concepts, thereby contributing to improved physical health and well-being of older people.

**Frailty** is a state of vulnerability, arising as a result of degradation of the physiological reserve and the ability to maintain homeostasis. In this state, some deleterious factors that are generally considered to be minor can trigger a significant decrease in health status. Frailty can be assessed through the Fried phenotype model or the frailty index (FI), including its electronic version eFI, by utilizing routine medical data. The Fried phenotype model, which focuses on physical frailty as a clinical pre-disability syndrome [5], identifies frailty when three of the following five criteria are met: weak grip strength, slow walking speed, low physical activity or decreased mobility, exhaustion, and unintentional weight loss [6]. The frailty index, also known as the cumulative deficit model, examines the accumulation of various “deficits” (symptoms, signs, diseases, and disabilities) [7] at the level of different systems [1,8,9]. In addition to those mentioned above, there are some other valuable tools in the identification and management of frailty in older adults, each with its strengths depending on the context in which they are used. Some frailty assessment tools and their particularities are summarized in Table S1.

#### 1.2.1. Prevalence

It has been estimated that approximately 25–50% of people older than 85 are frail [10]. It is important to specify that up to 75% of these people may not present severe frailty, highlighting the importance of studying the etiology of frailty and methods for its prevention [10], as well as frailty evaluation methods [11]. A study published in 2018 reported that, globally, 3.5–27.3% of non-institutionalized adults were frail [12].

#### 1.2.2. Risk Factors

Various risk factors for frailty have been proposed (Figure S1), including advanced age, male sex, low body mass index, lack of physical activity, excess medication, smoking, alcoholic beverages, malnutrition, and lack of vitamin D. However, regardless of the level of physical activity, aging itself is a factor for physiological frailty [2].

Older adult individuals become more vulnerable when they have concomitant chronic diseases, are exposed to acute infections, or are prone to falls [2]. Patients with diabetes, hearing problems, or depression seem to be more prone to frailty compared to those with cardiovascular or ophthalmological problems [8,12]. Studies have demonstrated that increases in the C-reactive protein (CRP) level, fibrinogen, and overall inflammatory activity, as well as increased blood cortisol, are also risk factors for frailty [13].

In addition to clinical, biological, and lifestyle factors, social criteria such as social isolation or loneliness [14] should be included in screening programs for frailty [13]. In this regard, there are studies [1,3,15] that reported a positive relationship between loneliness and worsening frailty, as well as a negative impact on recovery from the pre-frail or frail state. Loneliness is correlated with a lack of physical activity, representing a way in which the occurrence of frailty can be favored [16].

Frailty increases the risk of developing disability, the risk of hospitalization or institutionalization, and the risk of mortality [4,17,18]. An increased frailty index is associated with increased reporting of loneliness in older adults, as well as an increased risk of worsening loneliness over time [1,16].

#### 1.2.3. Classification

The Clinical Frailty Scale [19] is a commonly used frailty assessment tool based on clinical judgment and physical examination and allows classification in one of nine levels of frailty, from the “very fit” stage to the terminally ill stage.

Pre-frailty represents an intermediate state between frailty and robustness, with an increased risk for the transition to frailty. Individuals with this frailty prodrome are also exposed to adverse health effects, such as an increased risk of developing cardiovascular disease or depressive syndromes, or the worsening of pre-existing cognitive impairment [20].

Frailty is a clinical syndrome characterized by an increased vulnerability to seemingly unknown stressors [6] due to low physiological reserves, marked by a decline in the ability to maintain body homeostasis and multiple organ deficiency [10] as a result of the accumulation of age-related deficits.

Although distinct from multimorbidity, frailty overlaps with an advanced degree of disability and dependence on specialized help in the last decade of life [21].

Understanding the fluidity of frailty status between robust, pre-frail, and frail [22], it is important to consider potential interventions to improve the quality of life and maintain the functionality of older adults. Although it is closely related to age, frailty is not an inevitable and irreversible process, and it is imperative to understand the catalyst in the transition from robust to pre-frail or from pre-frail to frail in order to select the optimal population for slowing or preventing deterioration of functional status [23].

**Loneliness** is a natural phenomenon, being a feeling that appears in certain periods of life and affects people regardless of age, gender, or other socio-demographic characteristics. It should be noted that one may have a low number of social interactions but no negative emotions related to this fact; on the other hand, loneliness can also be felt by people who have social contacts and participate in collective activities [13,24].

Described as an experience of social isolation in the context of the inconsistency between a person’s desired versus real social relationships, loneliness implies a feeling of insufficient connection with others [25].

#### 1.2.4. Prevalence

The prevalence of loneliness worldwide varies widely; there are authors who have shown that 30% to 50% or even more of seniors are affected by loneliness and social isolation [24,26,27,28]. Estimated prevalences for different age groups and countries have relatively large variations, a fact that can be explained by the existence of difficulties in defining and evaluating the loneliness concept, the challenges of diagnostic and therapeutic approaches in different cultural and linguistic contexts, and the lack of validated tools or measuring instruments [24].

#### 1.2.5. Risk Factors

Two large categories of risk factors for loneliness have been described (Figure S2): individual factors (age, sex, race/ethnicity, education, employee/salaried status, financial situation, psychological factors/personality traits, health status, marital status, living arrangements—with family or in nursing homes—social network, and social activity) [29,30] and social factors (the living environment, facilities in the neighborhood, and the socio-economic and socio-cultural level of the country where the assessed person lives) [30].

#### 1.2.6. Classification

There is not a universally agreed-upon classification of loneliness. In the context of the complexity of this phenomenon, loneliness can be classified as follows: temporary or, on the contrary, persistent loneliness that can have consequences on physical and mental health [31]; situational or developmental loneliness (associated with life stages and transitions); digital loneliness (the result of excessive use of the Internet at the expense of face-to-face interaction and emotional connections achieved through socializing) [31,32]; emotional loneliness (inadequate emotional connections and lack of emotional intimacy and emotional support) [13]; interpersonal loneliness, based on “a negatively experienced discrepancy between realized and desired interpersonal relationships” [33]; existential loneliness, based on the awareness that “a human being is fundamentally alone” [33]; subjective loneliness that refers to the personal experience of the individual (it may not correspond to the number of social connections); and objective loneliness (isolation) that can be measured and evaluated by observable factors, such as the number of interactions or social relationships, regardless of the individual’s subjective feelings [34,35]. Therefore, “social loneliness” is an objective, quantitative assessment, which reflects the number of social contacts made by a person [1,35]. Loneliness, the subjective phenomenon experienced by a person, related or not to social isolation, is a factor of interest due to the possible two-way relationship it has with frailty, with the hypothesis being a “withdrawal” syndrome resulting from a continuous deterioration (decrease) in vital energy or through a lack of interest or capacity in engaging in social contacts [20].

There are authors who underlined that “loneliness and isolation place people at risk of vulnerability or social frailty” [13]. Social frailty was defined as “a continuum of being at risk of losing, or having lost resources that are important for fulfilling one or more basic social needs during the life span” [36], or as “a state of increased vulnerability to the interactive back-and-forth of community including general resources, social resources, social behaviors, and needs” [37]. This term is part of the broad concept of frailty, along with physical and psychological frailty [37,38,39] which are closely related. Unlike physical frailty, social frailty encompasses aspects of loneliness and isolation, affecting mental and physical health. It seems that “frailty and both loneliness or social isolation frequently overlap, particularly in areas of high socioeconomic deprivation” [40].

Older people who feel lonely and socially isolated have certain characteristics in common. These include older age, single status, male gender, low education, and low income [41]. 

The distinction between emotional loneliness and social loneliness is critical [33] as these types of loneliness can have different impacts on physical frailty. While emotional loneliness mainly affects mental health and emotional well-being and can have a secondary impact on physical health thus indirectly contributing to frailty, social loneliness has a more direct impact on physical health (through reduced physical activity, poor nutrition, and lack of social support) [27,28,42]. This direct impact on physical functioning increases the risk of physical frailty. The highlighting of these particularities is essential for the optimal, targeted approach, with the aim of ensuring and maintaining the well-being of old people for as long as possible.

The bidirectional relationship between loneliness and frailty highlights the importance of early and ongoing interventions. As an argument of loneliness as a risk factor for frailty, the following stages can be listed: the feeling of being alone causes mental stress (and rapid cognitive impairment) [42]; in this context, the level of stress hormones increases, and the persistence of stress reactivity can represent a risk factor for different chronic diseases [43], based on the inflammatory phenomenon; and inflammation has been described as the turning point of physical decline through reduced mobility, reduced muscle mass, and the appearance of physical frailty. Depression, an important mediator between loneliness and cognitive decline [42], can reduce an individual’s ability to care for themselves, leading to physical frailty. Through behavior pathways (e.g., physical inactivity, poor diet, alcohol consumption, or smoking), pathophysiological mechanisms (e.g., inflammation and stress reactivity) [43], and cognitive impairment [42], loneliness can indirectly damage health and induce physical frailty.

On the other hand, there are arguments for frailty leading to loneliness, linked to the physical limitations of frail people. Decreased mobility, pain, and fear of falling will reduce social interactions, promote social withdrawal, and increase dependence on caregivers [28,39,44].

There are studies examining how frailty in combination with loneliness or social isolation is associated with morbidity and all-cause mortality [37,40]. Although it appears that physical frailty or physical decline generally precedes mental decline, and an improvement in physical frailty also leads to an improvement in loneliness, in older adults, it has been observed that the reverse relationship is not always true: an improvement in loneliness does not always lead to an improvement in (physical) frailty [4,16,40]. A meta-analysis found that socially isolated individuals had a 1.5 to 2 times higher risk of becoming frail compared to those who were socially active [45]. Moreover, social frailty has been shown to possibly precede physical frailty in older adults [37,39]. It seems that the optimal approach requires an intervention both at the social level and at the individual level [36,46]. Therefore, promoting social connections, encouraging participation in social activities, and developing adequate social support are key factors in maintaining the health and well-being of older people [13,36,40,46].

### 1.3. Objective

The frailty of older adults is potentially preventable, and, therefore, knowing the associated risk factors is an absolute necessity both for prevention and to slow down, stop, and even reverse the frailty process once it has set in. We present a narrative review based on the available literature focusing on frailty and loneliness or social isolation in the context of aging. We opted for the narrative review format as it enables us to synthesize findings from diverse methodologies and contexts, highlighting gaps and inconsistencies in the literature.

Our question research was “Is there a link between the loneliness and frailty in older people”?.

## 2. Materials and Methods

### 2.1. The Search Strategy

Although the frequency of frailty and loneliness is high, the mechanism by which loneliness influences the progression of frailty remains incompletely elucidated. To assess the link between frailty and loneliness, we conducted a search accessing Index Medicus and PubMed in September–December 2023 (Table 1).

The following search strategy was used: loneliness AND frailty AND (fulltext:(“1” OR “1”) AND mj:(“Frail Elderly” OR “Aging” OR “Geriatric Assessment” OR “Health of the Elderly” OR “Quality of Life” OR “Activities of Daily Living” OR “Primary Health Care” OR “Risk Factors” OR “Prevalence”) AND la:(“en”)) AND (year_cluster:[2013 TO 2023]).

The inclusion criterion was any study (either descriptive or analytic) enrolling adult patients over the age of 60 diagnosed with frailty (either using Clinical Diagnostic Criteria for frailty or other validated diagnostic scales or instruments) and/or loneliness. Diagnostic criteria for frailty included Clinical Diagnostic Criteria, Fried frailty phenotype, Short Physical Performance Battery (SPPB), Frailty Deficit Index (FDI), Tilburg Frailty Indicator (TFI), Clinical-Functional Vulnerability Index-20 (IVCF-20), Edmonton Frail Scale, or Comprehensive Geriatric Assessment (CGA). Loneliness assessments considered self-reported scales capturing feelings of social isolation, emotional distress, and lack of companionship. Only studies published in English from 2013 to 2023 were included.

Studies were excluded if they were in a language other than English, if the full text was not freely available, if they were published more than 10 years ago, or if the studies were on populational groups other than adults over 60 years old without frailty and/or loneliness. Studies not identified using this search strategy that were subsequently identified and met the mentioned inclusion criterion were also included in our study. We opted for the age threshold of 60 years to ensure relevance for the aged population, we decided to include several validated diagnostic criteria for frailty and loneliness to ensure comprehensive coverage, and we selected articles in English published in the last 10 years to maintain the relevance and feasibility of our study.

### 2.2. Study Appraisal

The first evaluation was made by reading the title and abstract. Titles and abstracts of all identified studies were independently screened by two reviewers, and studies clearly not meeting the inclusion criteria were excluded at this stage.

The subsequent evaluation of the remaining articles involved independent reading of the full text to determine eligibility for inclusion in our study. Duplicate articles were excluded. We took descriptive or analytic studies enrolling adult patients diagnosed with frailty and/or loneliness into consideration.

We considered data extraction related to the type of study and number of participants, assessment tools or models used for each process, various assessment criteria for frailty and loneliness (including physical functional status, thinking, nutrition, comorbidities, marital status, and problems related to social isolation or loneliness), and key findings. During data extraction, we took into account the fact that, in the literature, in order to implement intervention strategies that are as suitable as possible for the target groups, different methods for assessing loneliness and frailty have been proposed. Furthermore, loneliness and frailty have been classified over time according to various criteria. For instance, loneliness can be assessed via self-reported scales that capture feelings of social isolation, emotional distress, and lack of companionship. Regarding the risk factors that can also represent the starting point for intervention plans, loneliness can be addressed through increasing social support, increasing opportunities for social interaction, improving social skills, and socio-cognitive training [13]. Frailty, on the other hand, can be measured using multidimensional instruments that assess physical function, cognition, nutrition, and other relevant domains, such as the frailty index or frailty phenotype (Clinical Frailty Scale [19], simplified Clinical Frailty Scale [47], Edmonton Frail Scale (https://edmontonfrailscale.org/validation-scale-and-spread, accessed on 2 July 2024), FRAIL scale (https://www.bgs.org.uk/sites/default/files/content/attachment/2018-07-05/rockwood_cfs.pdf, accessed on 2 July 2024), INTER-FRAIL (https://agsjournals.onlinelibrary.wiley.com/doi/10.1111/jgs.13029, accessed on 2 July 2024) Prisma-7, Sherbrooke Postal Questionnaire (https://www2.gov.bc.ca/assets/gov/health/practitioner-pro/bc-guidelines/frailty-prisma7.pdf, accessed on 2 July 2024), Short Physical Performance Battery (https://geriatrictoolkit.missouri.edu/SPPB-Score-Tool.pdf, accessed on 2 July 2024), and Study of Osteoporotic Fractures Index (https://agingresearchbiobank.nia.nih.gov/studies/sof/, accessed on 2 July 2024) or newer scales, for example, Kihon’s list (http://jssf.umin.jp/pdf/Kihon%20Checklist.pdf, accessed on 2 July 2024) [23].

Taking all these aspects into account, we decided to evaluate any study (either descriptive or analytic) enrolling adult patients diagnosed with frailty—either using Clinical Diagnostic Criteria for frailty, Fried frailty phenotype assessment, the Short Physical Performance Battery (SPPB), the Frailty Deficit Index (FDI), the Tilburg Frailty Indicator (TFI), Clinical-Functional Vulnerability Index-20 (IVCF-20; instrumental daily living activity, cognition, mood, mobility, communication, and multiple comorbidities), the Edmonton Frail Scale, or the Comprehensive Geriatric Assessment (CGA)—and/or loneliness.

Aiming to identify common patterns and differences in how studies have assessed and addressed loneliness and frailty, we focused on assessment methods and key findings regarding the possible bidirectional relationship between loneliness and frailty. When available, we extracted quantitative data from the included studies (but some of the selected studies did not report quantitative data, representing one of the limitations of our research).

## 3. Results

Starting from the association between frailty and loneliness, 213 articles were selected. After reading the titles and abstracts, 74 studies were excluded. The full texts of all remaining articles were read, and two independent authors (E.B. and A.-C.G.) evaluated and included the eligible articles. Disagreements between the two reviewers (E.B. and A.-C.G.) were resolved through discussion, and if consensus could not be reached, a third reviewer (R.I.N.) was consulted. All different opinions were subsequently presented and discussed with all the other authors. Finally, only 18 studies were analyzed (Table S2).

Through examining the relationship between loneliness and frailty, our aim was to shed light on possible ways in which the subjective feeling of being alone or isolated may contribute to physical decline and increased vulnerability among older adults. We set out to explore the epidemiology of loneliness and frailty, risk factors and potential consequences, classification systems, and discussions of the impact of loneliness on frailty [10,48].

In one of the evaluated studies, a total of 2817 people aged ≥60 from the English Longitudinal Study of Aging were assessed through the Fried phenotype model, the frailty index, and the UCLA Loneliness Scale; the authors concluded that there is a significant correlation between loneliness and frailty, “elderly people who experience high levels of loneliness are at increased risk of becoming physically frail” [1], and frailty can lead to increased social isolation [1]. In a prospective interventional study conducted by Ozic et al. [2], it was confirmed that “frailty in the elderly is related to increasing age, the status of single living, reduced functional capacity, quality of life, risk of falls and bone fractures” [2]. Moreover, the authors underlined that “with the progression of age psychological frailty of elderly persons also increases”. In this study, intervention targeting loneliness led to an important reduction in frailty scores (Tilburg Frailty Indicator and Groningen Activity Restriction Scale) over a 12-month period [2].

A systematic review, which included five reviews with a total of 227,381 participants, evaluated 26 questionnaires and eight frailty indicators, primarily among older adults living in the community [9]. Following the evaluation, the following were found: 1. the frailty index had adequate diagnostic and predictive capabilities; 2. gait speed showed high sensitivity, moderate specificity, and strong predictive strength for future disability in daily living activities; and 3. the Tilburg Frailty Index was reliable and valid for frailty screening, but did not assess diagnostic accuracy. Overall, low physical activity emerged as a key predictor of future decline in daily functioning [9] for physical but also emotional dependence followed by isolation and loneliness.

### 3.1. Chronic Health Conditions

Several studies have examined the prevalence and incidence of loneliness and frailty among older adults. For example, a study [49] showed that the prevalence of frailty among community-dwelling older adults ranges from 4% to 59%, depending on the diagnostic criteria used, and up to 66% of older adults suffer from at least two chronic conditions [49].

According to Sha et al. [16], there exists a reciprocal relationship between frailty and loneliness, and they share a pro-inflammatory phenotype [50]; however, the initial effect of frailty on subsequent loneliness is greater than if the events occurred in the opposite direction. This suggests that physical health has a greater influence on mental health than vice versa, which is in agreement with a previous study by Luo et al. [51]. The same study [16] showed that the effects of loneliness on frailty impairment in a six-year cohort were stronger than those in the shorter three-year cohorts.

The association of frailty with chronic diseases—with up to 66% of older adults suffering from at least two chronic conditions [49]—calls for more effective prevention strategies to reduce potential risks, knowing that the existence of comorbidities limits the social interactions and physical independence of the seniors and may represent a risk factor for loneliness. This co-association of several emerging factors with basic diseases of the individual is called multimorbidity, and there are studies that attest to the cause–effect relationship between this condition and the association of frailty with mortality [52]. Damluji et al. [53] found that pre-frailty and physical frailty phenotypes were associated with a high risk of major adverse cardiovascular events and mortality, despite rigorous control of cardiovascular risk factors, during 6 years of follow-up. Their conclusion was that subjects with frailty were older, female, and belonged to an ethnic minority. The authors concluded that efforts should be made to evaluate the efficiency and effectiveness of programs to maintain active status through promoting physical exercise, adequate nutrition, and cognitive training to prevent or even reverse frailty in patients with cardiovascular risk [53]. In another study in which the data of 9450 participants with 14 chronic somatic conditions and mental disorders were analyzed [52], the following was shown: (1) multimorbidity was associated with worsening transitions in frailty states among older US adults; (2) there were variations in the relationships between osteoarticular, neuropsychiatric, and cardiometabolic diseases and complex multimorbidity and frailty transitions; (3) there was a lower risk of worsening frailty in older patients with osteoarticular diseases compared to older patients with cardiometabolic diseases, neuropsychiatric–sensory disorders, and complex multimorbidity; and (4) the identification of multimorbidity patterns and their correlation with different stages of frailty can help to optimize the therapeutic intervention in order to improve the state of frailty among older adults [52].

### 3.2. Gender Differences

Sha et al. have reported that greater loneliness was related to an increased risk of worsening frailty and remaining frail [18], and it seems that older men with a high level of loneliness had a worse degradation of clinical status through frailty compared to older women [18].

Although frailty is chronologically and biologically related to age, occurs with a higher prevalence in women than in men [49], and is usually associated with chronic diseases [54], there is great heterogeneity in terms of the prevalence and degree of impairment associated with frailty in groups of people who fall under the same clinical–biological criteria. The higher prevalence of frailty among women may be explained by having, on average, lower body mass and lower muscle strength. It was argued that frailty is better tolerated by women than men and that women have lower mortality rates at any level or age of frailty [49]. In addition, it should be taken into account that women live longer on average than men, and the association of increased age with frailty has been demonstrated in various studies [49,54].

### 3.3. Social Vulnerability and Loneliness

The extrinsic variables, mainly included under the name of social vulnerability (relating to socio-economic level, social relations, and family support), in the context of their decrease, have been associated with greater frailty and an increased rate of in-hospital death [13]. Maltby et al. [20] described that loneliness and social isolation, compared to having an extensive social network, predict the frailty index for a period of more than four years both for basic frailty and other variables (e.g., age, socio-economic status, educational abilities, depressive symptoms, and smoking). The authors emphasize that measuring the multidimensional aspects of social isolation, loneliness, and frailty is essential for identifying those individuals who need intervention [20].

According to a 2020 report by the National Health and Aging Trends Study, in the United States, a significant number of older adults (approximately 7.7 million people) experienced loneliness and social isolation [55]. Before the Coronavirus Disease 2019 (COVID-19) pandemic, about 24% of adults aged 65 and older living in the community were socially isolated, and 4% of them were severely isolated [55]. Some studies have pointed out that approximately one in four community-dwelling adults are socially isolated. It seems that, among older adults, risk factors for social isolation include male gender, lower income, and lower educational attainment [52,55,56]. According to a study based on data from the U.S. Health and Retirement Study, 43% of Americans aged 60 and older reported feeling lonely [15]. Additionally, a survey conducted by The American Association of Retired Persons (AARP) found that 35% of adults over the age of 45 feel lonely [57]. In Europe, around 20% of older people experience loneliness. This means that 1 in 5 older people feel a sense of isolation and a lack of social connection. The percentage varies by country, with higher levels of loneliness in countries such as Sweden and Norway (over 25%) and lower levels in Spain and Greece (under 15%), underscoring the scale of the problem and the significant social impact on older people. It is important to pay attention to this aspect and develop prevention and intervention strategies to combat loneliness and promote well-being among the older population [41].

Loneliness influences the involvement in physical and social activities, which functions as a mediator in the loneliness–frailty relationship, with decreasing involvement leading to the progression of frailty [58]. Being often correlated with social isolation, loneliness can reduce social interactions. The lack of a network to provide physical and mental support can determine difficulties in accessing different types of services (including medical services). Physical inactivity causes decreased mobility and osteo-articular damage, decreased cardiovascular fitness, and functional decline of the entire body. Moreover, there may be a lack of motivation to live, a decrease in self-care motivation and physical inability for it, the appearance of mental problems, social withdrawal, the loss of self-esteem, or behavioral changes due to inadequate nutrition or the abuse of various substances (causing or exacerbating neurological disorders). In this way, a vicious circle through which loneliness generates frailty (that can be amplified on a physical, cognitive, and emotional level) and vice versa is created.

The bidirectional relationship between frailty and loneliness was assessed in a study [18] that followed the transition of frailty in two cohorts after 2008, followed up in 2011 and 2014. Tendencies to remain in a frailty state were associated with increased levels of loneliness observed over a period of three years: compared to patients who never felt lonely, those who often felt lonely were less likely to remain in the robust or pre-frail state. Additionally, in the group following a worsening of health, loneliness was a risk factor, such that increased levels of loneliness were associated with increased frailty over time [18].

A systematic review that included 21 randomized controlled trials and examined data from 5275 participants confirmed that frailty is modifiable, with interventions showing positive effects in different populations, including very old people in hospitals or long-term care [59]. The effectiveness of combining physical exercise with nutrition and cognitive training has been highlighted; interventions such as exercise without group support and multidisciplinary care without specific interventions were less effective, although they still improved independent function. This study included economic evaluations that also suggested that personalized frailty management, especially within the community, is also cost-effective [59].

### 3.4. Conjugal Life and Affective and/or Sexual Experiences

According to Hanlon et al., social isolation and/or loneliness are risk factors for increased hospitalization at all levels of frailty [3]; moreover, it seems that the risk of loneliness is more pronounced in those with a robust or pre-frail status [3,18]. Having a conjugal life, living with a partner, and being sexually active are factors correlated with robustness. Older people involved in couple activities seem to have a high quality of life based on common concerns for well-being, maintaining their social role, sharing common interests, and spending time practicing physical activity or having a healthy lifestyle. Companionship, trust, affection, and complicity are expressed in a particular way by older adults and seem to play an essential role in maintaining psychosocial identity and preserving interest in everyday life [60]. A possible problem for older couples is represented by the fact that with advancing age comes the difficulty of managing age-specific conditions or comorbidities. Sometimes, it is necessary for one of the members of the couple to take on the role of caregiver of the life partner, which can represent a serious problem for older individuals, especially for those who live alone (either because they have no offspring, or because their offspring live at long distances). It seems that female caregivers, caregivers with cognitive deterioration, and individuals who need help in carrying out household activities are the most prone to rapid deterioration and require a lot of attention from relevant healthcare providers [44].

## 4. Discussion

The aging process and the association of chronic diseases with advanced age are conditions that necessitate careful evaluation and investigation of health statuses. The prevalence of many diseases is increased in older populations. Surviving longer with diseases can be a factor in reducing functionality, implicitly leading to a limitation of the activities of people who are in their later years who then become dependent on their family and social care.

Aging causes not only numerous changes in the body and the appearance of chronic diseases but also socio-economic changes that represent a challenge both for old individuals and for society.

Considering the possible arguments for the bidirectional relationship between loneliness and frailty, it is obvious the importance of actively looking for these phenomena in an older adult population. Addressing loneliness and frailty in older people simultaneously, when issues related to loneliness, frailty, or both have been identified, can improve their quality of life, reduce health care costs, and promote healthier aging. Optimal interventions should be tailored to the individual’s stage of frailty and should include a combination of physical, psychological, and social strategies.

In primary healthcare, the proactive identification of loneliness, especially for the aged population, should be a priority and should be constantly carried out at every medical visit, regardless of the physical health status of the patient, in order to ensure optimal interventions at the right time [3].

After assessing the patient’s degree of frailty and loneliness and/or social isolation, the attending physician can offer patient-centered care, which could lead to more optimistic results and avoid the worsening of their condition [14].

The loss of autonomy in aging individuals is associated with needs of a medical, social, and psycho-affective nature [61], which must be evaluated according to the criteria for classification in degrees of dependence in different types of assessment grids for older adults [62,63]. In Romania, the national grid for assessing the needs of older persons was revised in 2023, ensuring the complete assessment of dependent older people (www.mmuncii.ro/j33/images/Documente/MMPS/Rapoarte_si_studii_MMPS/DPSS/2022_Substantiation_Study_for_LTC_Strategy_2023-2030_EN.pdf, accessed on 2 July 2024). Thus, seniors can be provided with social and medical services adapted to their individual needs. Society can support seniors who need assistance with personal care not only through ensuring access to professional care services but also by creating help centers where the older people can benefit from the provision of basic needs ranging from accommodation, care, and feeding to psychological support, social interaction, occupational therapy, religious/spiritual services, or legal counseling [41,64,65].

In the early stages of frailty, the progression of frailty could be reduced by encouraging participation in different types of social activities, such as volunteering, and access to senior centers or support groups that encourage physical activity and mental stimulation. In this way, physical activity, psychiatric support, social involvement, and emotional support are ensured. Social prescribing, physical activity programs, and mental health support are essential strategies for pre-frail and early frail status.

In moderate stages of frailty, in addition to physical activity programs that address physical mobility but also promote social interaction, ensuring adequate nutrition and providing home support and monitoring devices are helpful. The intervention of the integrated care team is essential to meet the needs of older adults with moderate frailty, to ensure the education of patients and caregivers regarding the maintenance of social interaction, and to provide logistic support (furnishing the home, performing daily tasks, and obtaining the necessary devices for movement or communication).

In advanced stages of frailty, a multidisciplinary approach is required to meet the medical, psychological, and social needs of frail older people and their caregivers. Towards the end of life, the palliative care team can provide comfort and reduce feelings of sadness, hopelessness, or isolation and loneliness. Palliative care services are representative of multidisciplinary stakeholders in the healthcare context [61,64,65] and are the most suitable to develop current and terminal medical care approaches, being one of the few services that can offer a holistic approach to the patient and those close to them [64,65]. Frailty services should be tailored to the needs and preferences of patients at their end of life [65], especially because older adults who live in specialized institutions, who are far from their friends and family, are more likely to experience loneliness, compared to individuals living in the community.

Social isolation and loneliness are risk factors for chronic diseases but can also be their consequence [61]. There exists a two-way path between them, and social isolation and loneliness can also dissuade an individual from fighting against disease and weakness, reducing the chances of the effectiveness of therapeutic interventions and worsening their status. Approaching the palliative rehabilitation care model adapted to an aging population with chronic non-oncological conditions can represent an opportunity to maintain interest in involvement in daily activities, thus maintaining their motivation to live as actively as possible in the last part of their lives [61].

Everyone in the latter part of their life is at risk of becoming frail [14]. Nevertheless, to the point where it becomes a pre-death phase, frailty is potentially preventable [14], and there is a belief that it could even be reversed if there is an early screening process followed by appropriate interventions [66].

Through our research, we aimed to study and understand the types of loneliness and their impact on the physical, cognitive, and emotional health of the senior population. The final goal is to increase the effectiveness of clinical practice, the orientation of targeted interventions, and the application of preventive strategies and public health policies aimed at mitigating loneliness and associated health risks. The significance of this study lies in its potential to provide a more holistic and detailed perspective on loneliness and frailty, thus contributing to improving the mental and physical well-being of this populational group.

The primary limitation of our study arises from the design of the included studies and their heterogeneity. Other potential limitations are language, publication and selection biases, data restriction, and also peculiarities in term definition and evaluation tools. We selected only studies published in English; this language restriction limited the generalizability of the results. Furthermore, we included only freely available full-text articles, which may exclude some high-quality studies that are not freely available; in addition, limiting the research to materials published between 2013 and 2023 may exclude earlier or more recent research that could provide valuable information about the relationship between loneliness and frailty. Another source of potential bias is that, although the initial screening and full-text review process was performed independently by two of the authors, this process may be subject to human error and subjective judgment, leading to the unintentional exclusion of relevant studies. Two more aspects are worth mentioning: the peculiarities of assessment tools for frailty and loneliness and the differences in the use of definitions and terminologies and the reported data, aspects that can conduct to heterogeneity in methods and outcomes, make it difficult to compare the results of different studies as well as to synthesize their findings. In this context, no final resolution can be postulated. The biological, psychological, and social mechanisms that potentially connect frailty and loneliness could be explored through longitudinal and/or interventional studies involving populations from different socio-economic backgrounds. Utilizing standardized and validated assessment tools to assess frailty and loneliness could guide future research efforts, ultimately strengthening the evidence for the bidirectional relationship between frailty and loneliness in older persons.

## 5. Conclusions

Considering the increasing prevalence of frailty, its early identification through risk factor assessment (including loneliness and/or social isolation) must become a standard of care for older patients. Loneliness can be seen as both a risk factor and a consequence of frailty, and it should be evaluated in the individual context of each old person. Taking into account the patient’s degree of frailty and their peculiarities, the care team can provide patient-centered care, which may lead to more optimistic outcomes, avoid the worsening of medical conditions, and ensure peaceful and active aging for older adults.

## Figures and Tables

**Table 1:** The search strategy summary.

Criteria	
Date of search	September–December 2023
Databases and other sources searched	Index Medicus
	PubMed
Search terms used	loneliness AND frailty AND (fulltext:(“1” OR “1”) AND mj:(“Frail Elderly” OR “Aging” OR “Geriatric Assessment” OR “Health of the Elderly” OR “Quality of Life” OR “Activities of Daily Living” OR “Primary Health Care” OR “Risk Factors” OR “Prevalence”))
Timeframe	2013–2023
Inclusion and exclusion criteria	Descriptive or analytic studies enrolling adult patients (over 60 years of age) diagnosed with frailty and/or loneliness; subsequently identified studies that met the mentioned inclusion criteria.
Selection process	Two independent authors (E.B. and A.-C.G.) evaluated and included the eligible articles.Different opinions were discussed with the other authors until a consensus was reached.

## Data Availability

Not applicable.

## Supplementary Materials

The following supporting information can be downloaded at https://www.mdpi.com/article/10.3390/geriatrics9050119/s1, Figure S1: Risk factors for frailty in older adults, Figure S2: Risk factors for loneliness in older adults; Table S1: Frailty assessment tools, Table S2: Summary of included studies.
