# Supplementary material for: Frailty and Loneliness in Older Adults: A Narrative Review
Source: Geriatrics (Basel). 2024 Sep 13;9(5):119. doi: 10.3390/geriatrics9050119 (PMC11417754; doi:10.3390/geriatrics9050119)
Supplement: Supplementary file 1 [file geriatrics-09-00119-s001.zip › 10.08 Table S1 Assesment Tools.pdf]

Table S1. **Frailty assessment tools**

Assessment tool	Description	Key findings
**1. Fried Phenotype Model**	- defines frailty as a clinical syndrome<br>- identifies frailty based on the presence of three or more of the following criteria: unintentional weight loss, self-reported exhaustion, weakness (grip strength), slow walking speed, and low physical activity.	- Focuses on physical components<br>- is a simple, easily applicable clinical tool<br>- emphasizes frailty as a distinct clinical entity.
**2. Frailty Index (FI)**	- based on the cumulative deficit model where frailty is measured by counting the number of health deficits a person has (e.g., symptoms, diseases, disabilities)<br>- is usually expressed as a ratio of the number of deficits present to the total number of deficits considered	- Comprehensive<br>- it includes a wide range of variables (physical, psychological, social)<br>- allows for a more nuanced assessment of frailty severity<br>- can be customized based on available data.
**3. Tilburg Frailty Indicator (TFI)**	- is a self-reported questionnaire that assesses frailty across three domains: physical, psychological, and social<br>- it includes questions on physical health, cognition, mental health, social relations, and living environment.	- Holistic, addressing multiple dimensions of frailty<br>- user-friendly, self-reported<br>- can be used in community settings<br>- useful for early identification of frailty.
**4. Groningen Activity Restriction Scale (GARS)**	- measures the degree of activity limitation in older adults<br>- it assesses a person's ability to perform basic and instrumental activities of daily living	- Focuses on functional abilities rather than frailty *per se*<br>- provides insights into the impact of frailty on daily life<br>- helps in planning interventions to maintain independence.
**5. Mortality Prediction Indexes (Lee, Schonberg)**	- estimate the mortality risk in older adults based on clinical factors<br>- the Lee Index considers factors like age, comorbidities, and functional status<br>- the Schonberg Index is tailored for use in community-dwelling older adults.	- Specifically designed for mortality risk prediction<br>- includes easily measurable clinical and functional variables<br>- useful for making decisions about care and interventions.
**6. Physical Frailty Phenotype (PFP) Scale**	- Similar to the Fried Phenotype<br>- measures frailty based on physical performance tests such as gait speed, grip strength, and balance<br>- it emphasizes the physical aspects of frailty.	- Objective, performance-based assessment<br>- focuses on physical function and mobility<br>- helps identify individuals at risk for adverse outcomes.
**7. FRAIL Scale**	- a simple screening tool that assesses frailty based on five criteria: Fatigue, Resistance (ability to climb stairs), Ambulation, Illnesses, and Loss of weight.	- Easy to administer, quick assessment.<br>- includes both physical and health-related criteria<br>- useful for initial frailty screening in clinical settings

**8. Variants of Frailty Index**	- modified versions of the original Frailty Index, tailored to specific populations or settings<br>- maintain the cumulative deficit approach but might focus on different sets of variables.	- Flexibility to adapt to different populations (e.g., dementia patients)<br>- allows for context-specific frailty assessment<br>- preserves the comprehensive nature of the original Frailty Index.
**9. Self-Reported Frailty Instrument**	- based on self-reports from individuals regarding their health and functional status.<br>- it typically includes questions about physical function, health perception, and daily activities.	- Empowers patients to self-assess their frailty<br>- is a simple, easily applicable tool<br>- useful in community-based settings or for large-scale screening.
**10. Clinical-Functional Vulnerability Index-20 (IVCF-20)**	- a 20-item questionnaire<br>- evaluates different dimensions of health including cognition, mood, communication, mobility, and activities of daily living	- Comprehensive<br>- is a simple, easily applicable clinical tool<br>- helps identify individuals at risk for adverse outcomes enabling targeted interventions.<br>- used in primary care and hospital settings to identify older adults at higher risk for adverse health outcomes (hospitalization, functional decline, or death).
**11. Edmonton Frail Scale (EFS)**	- a brief, multidimensional tool<br>- includes 11 items covering aspects related to: cognition, general health status, functional independence, social support, medication use, nutrition, mood, continence, and functional performance.	- Multidimensional<br>- simple to administer and interpret<br>- used in primary care, outpatient clinics, community settings<br>- good reliability and validity in identifying frailty, correlating well with other frailty assessment tools.<br>- effective in predicting adverse outcomes such as hospitalization, falls, and mortality.
**12. Comprehensive Geriatric Assessment (CGA)**	- multidimensional, interdisciplinary diagnostic tool<br>- covers aspects related to: physical health, mental health, functional status, social circumstances, and environmental factors.	- Holistic Approach<br>- involves assessments by a team of healthcare professionals<br>- effective in identifying unmet needs and addressing complex health issues, making it beneficial for frail older adults with multiple comorbidities.<br>- used in hospital settings, long-term care facilities, home care
