# Supplementary material for: Frailty and Loneliness in Older Adults: A Narrative Review
Source: Geriatrics (Basel). 2024 Sep 13;9(5):119. doi: 10.3390/geriatrics9050119 (PMC11417754; doi:10.3390/geriatrics9050119)
Supplement: Supplementary file 1 [file geriatrics-09-00119-s001.zip › 10.08. Table S2.pdf]

No. crt.	Reference	First author, year of publish	Study type/ number of participants	Type of process							Assessment model	
				Frailty (Evaluated criteria)					Social isolation /loneliness		Frailty	Loneliness
				Physical function	Cognition	Nutrition /ponderal state	Comorbidities	Marriage status /affective and/or sexual experiences	Social isolation	Loneliness		
1.	1.	Gale et al, 2018	Prospective study 6183 participants (2817 – wave 2)	x	x		x		x	x	Fried phenotype model Frailty index	The UCLA Loneliness Scale
2.	2.	Ozic et al, 2020	Prospective interventional study 410 participants	x	x	x	x	x	x	x	Tilburg Frailty Indicator Groningen Activity Restriction Scale	Tilburg Frailty Indicator Groningen Activity Restriction Scale
3.	3.	Hanlon et al, 2023	Longitudinal cohort 502 456 participants (UK Biobank)	x	x	x	x		x	x	Frailty phenotype Frailty index	Self-reported questionnaires
4.	8.	Wang et al, 2022	Systematic Review	x	x	x	x			x	Fried phenotype model Frailty index	
5.	9.	Apostolo et al, 2017	Scoping review, 5 reviews, 227 381 participants	x	x	x	x	x	x	x	Frailty index Variants of Frailty index	
6.	11.	Shi et al, 2020	Prospective study, 7033 participants	x	x	x	x		x		Frailty index Mortality prediction indexes (Lee, Schonberg)	
7.	17.	Sha et al, 2022	Prospective study	x	x	x	x		x	x	Physical Frailty Phenotype (PFP) scale	Single-question measurement of loneliness
8.	18.	Sha et al, 2020	Prospective study 16 840 participants	x	x	x	x		x	x	FRAIL Scale Frailty Index	Single-question measurement of loneliness
9.	22.	Siriwardhana et al, 2018	Systematic review and meta-analysis	x			x		x		Fried phenotype	
10.	41.	Fakoya et al, 2020	Scoping review	x	x		x	x	x	x		reported -as the primary outcome or -in the context of health outcomes
11.	44.	Santos-Orlandi et al, 2017	Cross-sectional study 40 participants	x	x	x	x	x			Fried phenotype	Geriatric Depression Scale
12.	49.	Ofori-Asenso et al, 2019	Systematic review and meta-analysis	x	x	x	x				Fried phenotype Frailty index	
13.	50.	Zhao et al, 2021	Cross-sectional study 740 participants	x	x	x	x	x	x		FRAIL scale	
14.	52.	Luo et al, 2023	Longitudinal study 9450 participants	x	x		x	x	x		Fried phenotype	
15.	53.	Damluji et al, 2021	Prospective cohort study 4656 participants	x	x	x	x	x	x		Fried phenotype	
16.	58.	Zhang et al, 2023	Longitudinal cohort study 634 participants	x	x		x		x	x		De Jong Gierveld Loneliness Scale Lubben Social Network Scale-6
17.	59.	Apostolo et al, 2018	Review, 21 randomized controlled trials, 5275 participants	x	x	x	x	x	x	x	Frailty index Variants of Frailty index	
18.	60.	Souza Junior et al, 2021	Sectional study 662 participants	x	x	x	x	x	x	x	Self-reported Frailty instrument	

Table S2. Summary of included studies
