# Supplementary figures and images for: Frailty and Loneliness in Older Adults: A Narrative Review
Source: Geriatrics (Basel). 2024 Sep 13;9(5):119. doi: 10.3390/geriatrics9050119 (PMC11417754; doi:10.3390/geriatrics9050119)

**Figure S1.** Risk factors for frailty in older adults

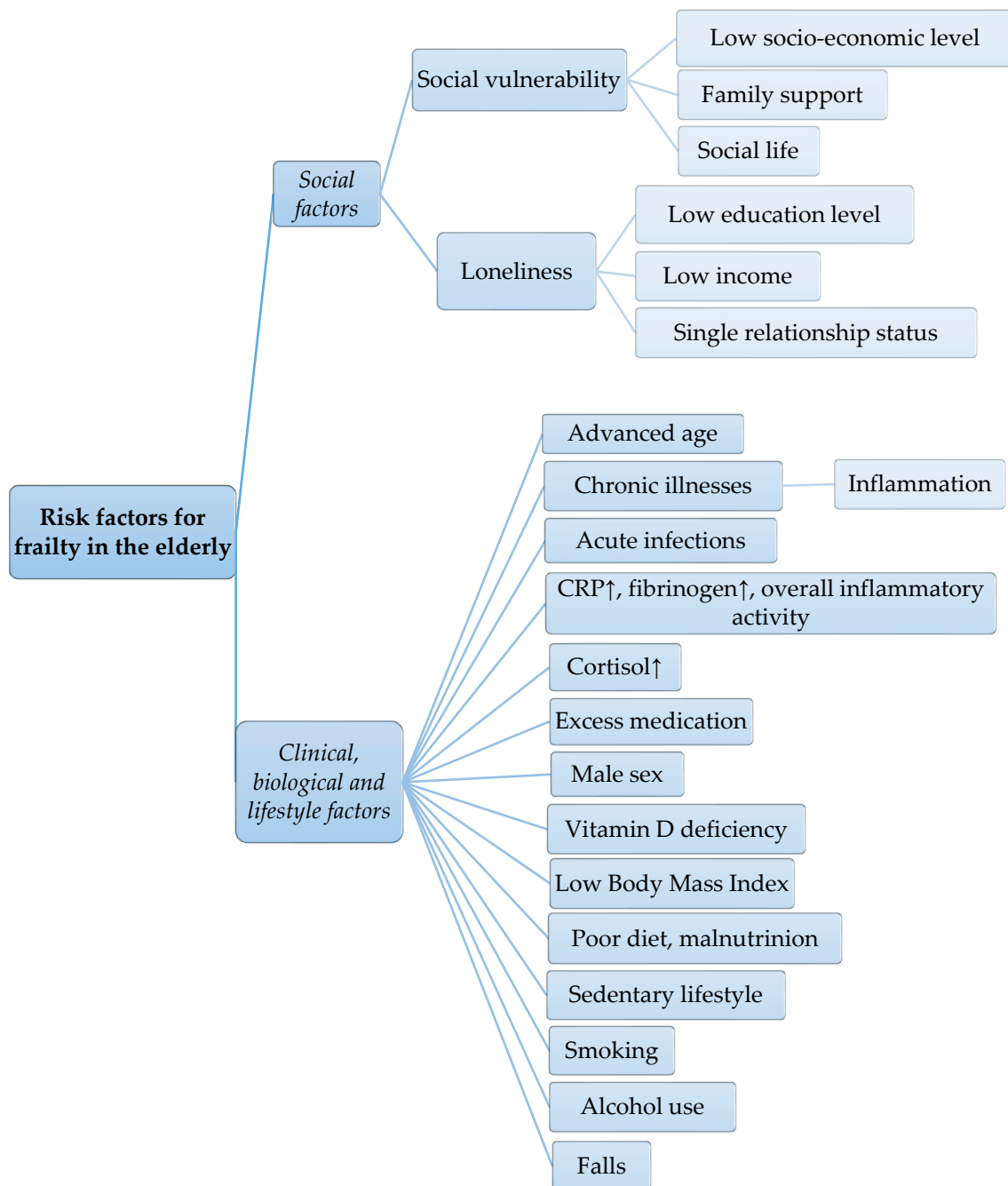

Supplement: Supplementary file 1 [file geriatrics-09-00119-s001.zip › FIGURE S1.pdf]

**Figure S2.** Risk factors for loneliness in older adults

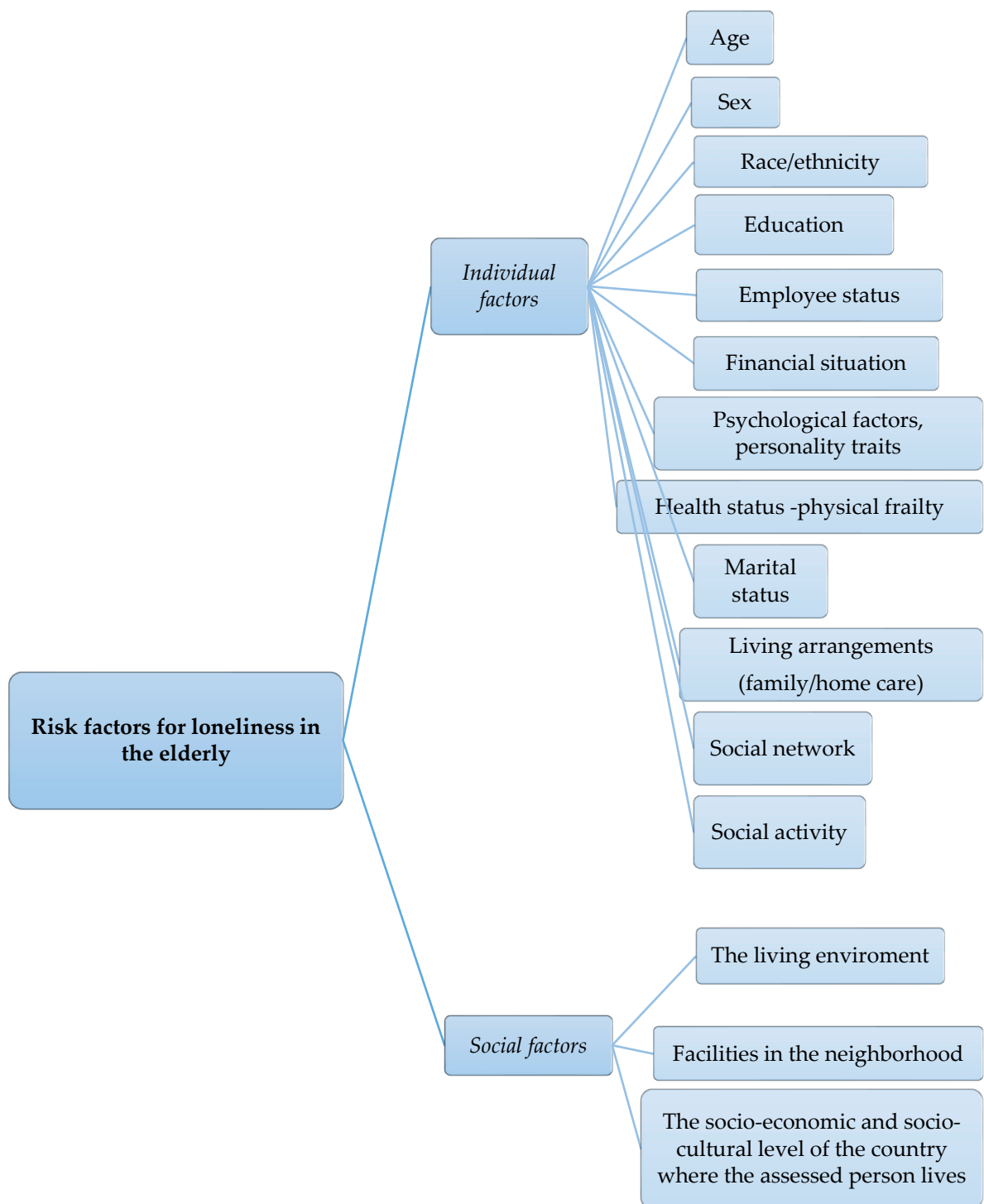

Supplement: Supplementary file 1 [file geriatrics-09-00119-s001.zip › FIGURE S2.pdf]
